# Supplementary material for: Human iPS cell–derived respiratory organoids as a model for respiratory syncytial virus infection
Source: Life Sci Alliance. 2025 Apr 22;8(7):e202402837. doi: 10.26508/lsa.202402837 (PMC12015132; doi:10.26508/lsa.202402837)
Supplement: Supplementary file 1 [file LSA-2024-02837_TableS1.docx]

| Gene name | Base Mean | log2 Fold Change | *P* value | *P*adj |
| --- | --- | --- | --- | --- |
| *OAS2* | 599.2990 | 10.4028 | 7.07E-49 | 2.35E-46 |
| *MX1* | 3566.4470 | 7.0923 | 1.99E-144 | 5.8E-141 |
| *IFIT3* | 1035.0422 | 5.6786 | 1.36E-83 | 8.85E-81 |
| *OAS1* | 878.1665 | 5.5326 | 3.43E-71 | 1.85E-68 |
| *IFIT2* | 965.8226 | 5.4184 | 1.19E-101 | 1.4E-98 |
| *IFI6* | 3983.6845 | 5.2769 | 1.07E-109 | 1.45E-106 |
| *RSAD2* | 108.1706 | 5.2415 | 8.7E-62 | 3.66E-59 |
| *ISG15* | 1768.9080 | 5.1880 | 5.59E-101 | 5.88E-98 |
| *HERC5* | 331.0904 | 4.9725 | 4.23E-116 | 8E-113 |
| *OAS3* | 1846.9721 | 4.9151 | 5.74E-114 | 9.88E-111 |
| *IFI27* | 584.2665 | 4.7501 | 2.15E-144 | 5.8E-141 |
| *IFIH1* | 538.6192 | 3.9381 | 3.76E-107 | 4.75E-104 |
| *DDX60* | 748.4332 | 3.5224 | 3.86E-146 | 1.82E-142 |
| *DDX58* | 977.6546 | 3.1534 | 1.57E-118 | 3.31E-115 |
