# Supplementary material for: Human iPS cell–derived respiratory organoids as a model for respiratory syncytial virus infection
Source: Life Sci Alliance. 2025 Apr 22;8(7):e202402837. doi: 10.26508/lsa.202402837 (PMC12015132; doi:10.26508/lsa.202402837)
Supplement: Supplementary file 2 [file LSA-2024-02837_TableS2.docx]

| Airway epithelial cells | ADH7,AQP1,CDH1,SEC14L3 |
| --- | --- |
| Airway goblet cells | AGR2,AQP5,CEACAM1,DMBT1,DUSP4,FXYD3,GALNT5,GALNT6,GGH,GOLPH3,GP2,IL19,LIPF,LTF,LYNX1,MSLN,MUC16,MUC4,MUC5B,NOS2,PIGR,QSOX1,SCGB3A1,SEC23B,SPDEF,TFF2 |
| Fibroblasts | COL3A1,COL5A2,DPT,FN1,GSN,LRP1,PDGFRA,TCF21 |
| Basal cells | ABI3BP,AQP3,DAPL1,GSTM2,HPGD,ICAM1,KRT14,KRT15,KRT5,PHLDA3,RPS18,SDC1 |
| Alveolar macrophages | ABCG1,CCL3,CD36,CLEC7A,CSF2RB,CXCL2,G0S2,GAL,GDA,GNGT2,GPNMB,IL18,IL1B,ITGAX,KLHDC4,MARCO,MCEMP1,MPP1,MRC1,OLR1,PLET1,S100A4,TLR2,TNFAIP2,TRIM25 |
| Ciliated cells | APPL2,ATP5MD,CCDC153,CCDC17,CCDC181,CCDC39,CYP2S1,FAM161A,FOXJ1,LRRC23,ODF3B,SCGB1A1,SEC14L3,SNTN,STK11,TMEM212,TSPAN19,TUBB4B |
| Club cells | AHR,ALDH1A1,BPIFA1,CTSE,CYP2E1,CYP4B1,ERN1,FOXM1,LEPR,MUC1,MUC4,MUC5B,RAB3D,SCGB1A1,SCGB3A1,SCGB3A2,SFTPA1,SFTPC,SFTPD,SYT2 |
| Immune system cells | ADGRE1,ARG1,BIRC5,CCL17,CCL18,CD14,CD163,CD19,CD22,CD3D,CD3E,CD3G,CD4,CD44,CD5,CD68,CD74,CD80,CD83,CD86,CD8A,CD8B,CDCA3,CDK1,CEBPE,CR2,CSF1R,EAF2,FCER2,FCGR3A,FLT3,GIMAP3P,HLA-DRA,HMMR,IGHM,IL2RA,ITGAE,ITGAM,ITGAX,LAX1,MS4A14,MZB1,NRP1,NUSAP1,PAX5,PRG2,PTPRC,SLPI,THBD,TNFRSF17,TRBC1,XCR1 |
| Endothelial cell | CD34,EGFL7,EMCN,ESAM,FLT1,KDR,MCAM,PECAM1,RAMP2,TEK,VWF |
| Epithelial cells | ANPEP,EPCAM,IL10,IL6R |
| Ionocytes | CFTR,CLCNKB,FOXI1,KCNMA1,SCGB1A1,SLC12A2,TFCP2L1 |
| Pulmonary alveolar type I cells | AGER,AKAP5,AQP3,AQP5,CCN2,CLDN18,CLIC5,COL4A3,COL4A4,CRLF1,CYP4B1,EGFL6,EMP2,FSTL3,GPRC5A,HOPX,ICAM1,IGFBP6,KRT7,MEX3B,MMP11,P2RX7,PDPN,PXDC1,RTKN2,SCNN1A,SCNN1B,SCNN1G,SEC14L3,SEMA3B,SEMA3E,SMARCA1,VEGFA |
| Pulmonary alveolar type II cells | ABCA3,ADGRF5,AGER,CD36,CD3G,CEBPA,CLDN18,CRLF1,CTNND1,CXCL2,CXCR2,DDX3Y,EGFL6,ETV5,GRK2,IL1B,INMT,IRX1,LAMP3,LPCAT1,LRG1,MUC1,NAPSA,NKX2.1,NRN1,PGC,PIGR,PPBP,PPP1R14C,RUNX3,S100G,SDC1,SFTA2,SFTPA1,SFTPB,SFTPC,SFTPD,SLC34A2,SOAT1 |
