# Supplementary material for: Human iPS cell–derived respiratory organoids as a model for respiratory syncytial virus infection
Source: Life Sci Alliance. 2025 Apr 22;8(7):e202402837. doi: 10.26508/lsa.202402837 (PMC12015132; doi:10.26508/lsa.202402837)
Supplement: Supplementary file 3 [file LSA-2024-02837_TableS3.docx]

| Antigen | Catalogue | Host | Company |
| --- | --- | --- | --- |
| Goat anti-Mouse IgG (H+L) Highly Cross-Adsorbed Secondary Antibody, Alexa Fluor™ 594 | A11032 | Goat | Thermo Fisher Scientific |
| Donkey anti-Rat IgG (H+L) Highly Cross-Adsorbed Secondary Antibody, Alexa Fluor™ 488 | A21208 | Donkey | Thermo Fisher Scientific |
| β-actin | A5441 | Mouse | Sigma-Aldrich |
| Acetylated α-tubulin | 66200-1-lg | Mouse | Proteintech |
| Respiratory Syncytial Virus F protein (MEDI-493) | AB02241-23.0 | Rabbit | Absolute Antibody |
